# Children’s altruistic behavior in context: The role of emotional responsiveness and culture

**DOI:** 10.1038/srep24089

**Published:** 2016-05-03

**Authors:** Purva Rajhans, Nicole Altvater-Mackensen, Amrisha Vaish, Tobias Grossmann

**Affiliations:** 1 Early Social Development Group, Max Planck Institute for Human Cognitive and Brain Sciences, Leipzig, Germany; 2 Department of Psychology, University of Virginia, Charlottesville, VA, USA

## Abstract

Altruistic behavior in humans is thought to have deep biological roots. Nonetheless, there is also evidence for considerable variation in altruistic behaviors among individuals and across cultures. Variability in altruistic behavior in adults has recently been related to individual differences in emotional responsiveness to fear in others. The current study examined the relation between emotional responsiveness (using eye-tracking) and altruistic behavior (using the Dictator Game) in 4 to 5-year-old children (N = 96) across cultures (India and Germany). The results revealed that increased altruistic behavior was associated with a greater responsiveness to fear faces (faster fixation), but not happy faces, in both cultures. This suggests that altruistic behavior is linked to our responsiveness to others in distress across cultures. Additionally, only among Indian children greater altruistic behavior was associated with greater sensitivity to context when responding to fearful faces. These findings further our understanding of the origins of altruism in humans by highlighting the importance of emotional processes and cultural context in the development of altruism.

Why humans engage in costly acts of altruism towards genetically unrelated individuals has been one of the most enduring and puzzling questions in biology and psychology^1^. The empirical work available now to address this question by tracing altruism’s phylogenetic and ontogenetic origins provides compelling evidence that altruistic behavior is deeply rooted in our biology. From the phylogenetic perspective, altruistic behavior is not unique to humans but also found in other animals including our closest living relatives, the chimpanzees^2^,^3^. Furthermore, from the ontogenetic perspective, altruistic behavior emerges very early in development during infancy, before socialization can culturally shape this behavior. For example, already at the young age of 14 months, infants help others in need^4^. Based on these comparative and developmental data, it has been suggested that it is in our nature to be altruists^5^.

Nonetheless, the tendency of humans to engage in altruism varies greatly across individuals. Indeed, there exist extreme cases with regard to this tendency, ranging from extremely prosocial kidney donors to highly antisocial psychopaths^6^,^7^. The study of these extreme cases not only informs the question of what contributes to individual differences in altruism but also sheds light on the basis of altruistic behavior more generally. In particular, emotional responsiveness to seeing others in distress (e.g., displaying fear or sadness) appears to be a key process related to altruistic tendencies, with kidney donors showing heightened and psychopaths showing decreased perceptual sensitivity to fear in others^7^,^8^. At the neural level, the amygdala shows enhanced responding to fearful faces in highly altruistic kidney donors and blunted responding in psychopaths^7^. Critically, these effects are specific to fearful faces as no such differences are evident in response to other emotions such as anger^7^. This work indicates that perceptual sensitivity to distress cues in others is associated with differences in altruistic behavior, in line with the view that a basic form of empathic responding is linked to altruism^9^. Indeed, from very early in development, empathic responding is a key affective ability underlying prosocial behavior^10^. For example, the degree to which young children experience empathic concern for another person in distress has been shown to correlate with helping and comforting behaviors directed at this person^11^,^12^, pointing towards an early developing link between empathy and prosocial behavior.

Importantly, while altruism rather than self-interest dominates social behavior across cultures, the degree of altruism varies considerably across cultures^13^,^14^. Studies of human altruism using economic games such as the dictator game – in which one person is allotted a divisible resource (usually money) and can then ‘dictate’ how much of that resource she is willing to give to another person – show that cultural differences in economic organization and the structure of social interactions explain a substantial portion of the behavioral variation in altruism across cultures^13^. Specifically, the greater the degree of market integration and the payoffs to cooperation with strangers in everyday life, the higher the level of altruism expressed in the dictator game. Interestingly, this is probably why, counter to intuition, altruistic behavior in such economic games is usually higher in individualistic than in collectivistic cultures^13^. Indeed, recent developmental research suggests cross-cultural differences along these lines in the emergences of children’s fairness norms^15^.

Furthermore, there is mounting evidence that perceptual processes are influenced by culture^16^. One body of work suggests that when viewing a visual scene, Westerners generally engage in context-independent and analytic perceptual processes by selectively focusing on salient objects independently of their context, whereas Asians typically engage in context-dependent and holistic perceptual processes by attending to the relation between the object and the context in which the object is placed^17^. For example, Westerners focused on foreground objects faster than Asian adults, and then continued to look at the focal object for longer^18^. These cultural differences in visual perceptual processing between Asians and Westerners first develop around 5 years of age^17^, suggesting that cultural experience and learning play a role in the emergence of such biases. It is thus plausible that cultural learning may also play a role in whether and how context affects responsiveness to emotions in others.

Surprisingly, the link between altruism and perceptual sensitivity to fear in others has thus far only been examined in adults in Western cultures^19^. It is therefore unclear whether this link exists in children and how cultural factors affect this purportedly hard-wired link between perceptual sensitivity to others’ distress and altruistic tendencies. Addressing these questions is particularly important because it can shed light on the psychology underlying altruistic behavior by allowing for a closer look at the emotional, developmental, and cultural origins of human prosociality.

In the current study, we therefore examined the relations between emotional responsiveness and altruistic behavior in 4- to 5-year-old children in India and Germany. Altruistic behavior was examined through the dictator game using stickers as resources. Prior to the dictator game, children participated in an eye-tracking task in which their responses to fearful and happy facial expressions, presented either in isolation or in the context of several neutral facial expressions, were examined. We tested three main hypotheses. First, if emotional responsiveness indeed plays an important role in accounting for differences in altruistic behavior, then an association between the two should exist already in childhood. More specifically, based on the prior work with adults, we predicted that the link should only occur with respect to responsiveness to fearful faces but not to happy facial expressions, and that greater sensitivity to fearful faces as indexed by faster orienting in the eye-tracking task should be associated with greater altruism in the Dictator Game. Faster orienting to fearful faces appears to be a particularly relevant measure, because the brain processes that have been found to correlate with individual differences in prosocial behavior and emotional face processing have been implicated in effective attentional orienting to fear^6^,^7^. Second, if the predicted link exists in childhood and can be considered a hallmark of human altruism, then it should be seen across cultures. Third, given existing evidence for overall differences in perceptual processing between Westerners and Asians^16^,^17^,^18^, there might also be culture-specific patterns such that Indian children may show a greater sensitivity to context when processing facial expressions and that this may in turn relate to their altruistic behavior. More specifically, Westerners tend to engage in context-independent and analytic perceptual processes by selectively focusing on salient objects independently of their context, whereas Asians typically engage in context-dependent and holistic perceptual processes by attending to the relation between the object and the context in which the object is placed^17^. For example, Westerners focused on foreground objects faster than Asian adults, and then continued to look at the focal object for longer^18^. These cultural differences in visual perceptual processing between Asians and Westerners first develop around 5 years of age^17^, suggesting that cultural experience and learning play a role in the emergence of such biases. It is thus plausible that cultural learning may also play a role in whether and how context affects responsiveness to emotions in others.

## Results

**Figure 1:**
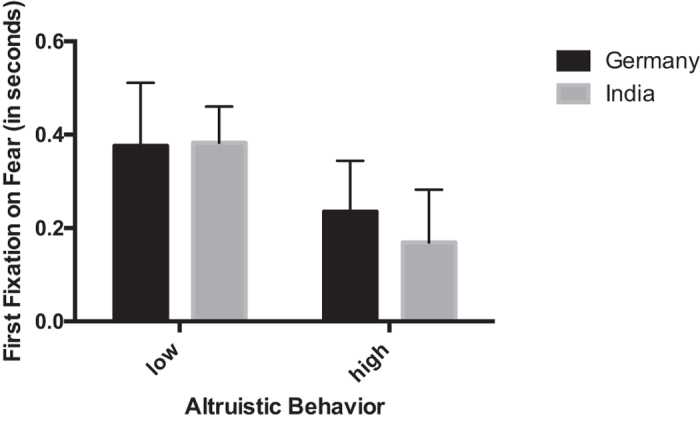
This figure shows the latency to the first fixation on fearful facial expressions in Task 1, where emotional faces were presented without a context, for children that show either low or high altruistic behavior in the Dictator Game separately for Germany and India.

**Figure 2:**
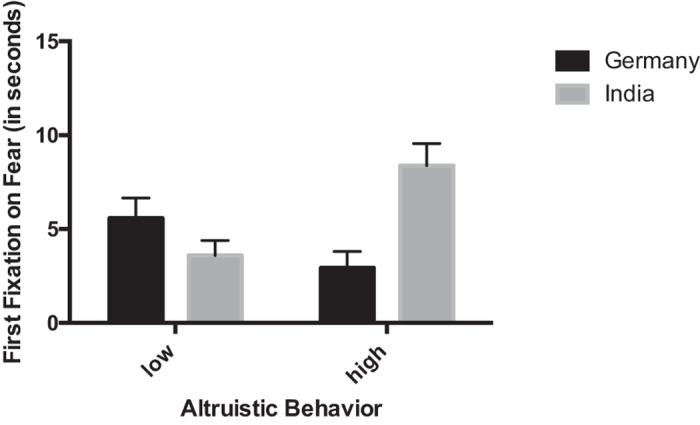
This figure shows the latency to the first fixation on fearful facial expressions in Task 2, where emotional faces were presented in the context of neutral facial expressions, for children that show either low or high altruistic behavior in the Dictator Game separately for Germany and India.

We conducted an omnibus repeated measures ANOVA for the latency to the first fixation on the emotional target face with emotion (happy, fearful) and context (without context [Task 1], with context [Task 2]) as within-subjects factors, and culture (Germany, India) and altruistic behavior (low [0 or 1 sticker] versus high [2 or more stickers] based on a mean split: *Mean = *1.33, *SE = *0.12) as between-subjects factors. Critically, there was no main effect of culture as a between-subjects factors on the latency to the first fixation on the emotional target faces measure, *F* (1, 91) = 0.5, *p* = 0.48, allowing us to rule out that there were overall cultural differences in the way in which children orient to faces in these two experiments. This analysis revealed a four-way interaction between emotion, context, culture, and altruistic behavior: *F* (1, 91) = 7.64, *p* = 0.007, partial *η*^*2*^ = 0.077. Follow-up repeated measures ANOVAs conducted separately for fear and happiness showed that a three-way interaction between context, culture, and altruistic behavior only existed for fear, *F* (1, 92) = 14.338, *p* = 0.0002, partial *η*^*2*^ = 0.135, but not for happiness, *F* (1, 92) = 0.32, *p* = 0.859, suggesting that this interaction effect is specific to fear. Further analysis to examine this interaction carried out separately for the two cultures revealed that for both the Indian and the German children there was a main effect of altruistic behavior on children’s processing of fearful facial expressions (Germany: *F* (1, 41) = 4.144, *p* = 0.048, partial *η*^*2*^ = 0.092; India: *F* (1,51) = 9.605, *p* = 0.003, partial *η*^*2*^ = 0.158). Specifically, as shown in Fig. 1, children who were more altruistic were faster to fixate on fearful faces presented without context (Germany: [low altruistic behavior] *M* = 0.37 seconds, *SE* = 0.13, [high altruistic behavior] *M* = 0.24, *SE* = 0.11; India: [low altruistic behavior] *M* = 0.38, *SE* = 0.08, [high altruistic behavior] *M* = 0.17, *SE* = 0.11). Our analysis further revealed that only among Indian children there was an interaction between altruistic behavior and context, *F* (1, 51) = 13.073, *p* = 0.001, partial *η*^*2*^ = 0.204. Specifically, as shown in Fig. 2, Indian children who were more altruistic were slower to fixate on fearful faces when these were presented in the context of neutral faces ([low altruistic behavior] *M* = 3.59, *SE* = 0.81, [high altruistic behavior] *M* = 8.38, *SE* = 1.17). In other words, Indian children who were more strongly influenced (slowed down) by context showed greater altruistic behavior.

## Discussion

We examined the relation between responsiveness to emotional faces (measured using eye-tracking) and altruistic behavior in the Dictator Game in children across cultures. Our results yielded three main findings. First, already in childhood, responsiveness to emotional faces is associated with differences in altruistic behavior. Specifically, similar to adults, children’s selective responsiveness to fearful faces as indexed by faster orienting in the eye-tracking task was associated with greater altruism (sharing more stickers) in the Dictator Game. Second, this link between emotional responsiveness to fear and altruistic behavior existed in children of both cultures. Third, there were also culture-specific patterns, especially in children’s processing of fearful faces in context. Namely, only among Indian children, those children who were slower to orient to fearful faces presented in the context of neutral faces (indexing a greater sensitivity to context) showed greater altruistic behavior in the Dictator Game. Taken together, our data suggest that across cultures and from early in development, altruistic behavior is tightly linked to our responsiveness to emotional signals of distress in others.

To our knowledge, the current findings are the first to provide direct developmental evidence for the existence of a link between emotional responsiveness to fear in others and altruistic tendencies. Such an extension of existing work with adults^8^ along a developmental dimension is important, because it suggests that responsiveness to fear in others might actually reflect a critical mechanism involved in the emergence of altruistic behavior. Furthermore, the current data point to emotional responsiveness to distress as a vital source for contributing to variability in the tendency of humans to engage in acts of altruism. Indeed, we may speculate based on these data that the striking differences in altruistic behavior observed between kidney donors on the one hand and psychopaths on the other hand in adulthood^7^ might have a strong developmental component rooted in differences in the responsiveness to others in distress^10^,^20^. The current study provides a novel way to measure and link responsiveness to emotions in others to altruism, and may thus offer a new tool to examine and predict altruistic behavior in childhood and beyond. With respect to future studies it will be important to extend this approach by looking at other emotional expressions such as sadness and pain that have been shown to elicit prosocial behavior in children^21^. Indeed, sadness or pain may even elicit greater prosocial behavior than fear, which may instead or in addition elicit withdrawal behavior. To include these emotional expressions in future work will help to determine whether the effects observed for fearful expressions in the current study generalize to other empathy-inducing expressions or are specific to fear.

Our data further revealed that increased altruistic behavior is associated with greater responsiveness (faster orienting) to fearful faces in Indian and German children. This is in general agreement with prior cross-cultural work that has identified similar influences on and patterns of prosocial behavior in German and Indian children at 2 years of age^22^. However, while Kärtner, *et al*.^22^ found parents’ socialization goals concerning interpersonal responsiveness (obedience) to play an important role in fostering prosocial behavior across cultures, our data indicate that intrinsic processes (such as the child’s propensity to respond to distress cues in others) are also critical. This increased focus on intrinsic and culture-independent factors to explain the emergence and variability of prosocial behavior in childhood is in line with recent proposals that (a) view empathic concern as a key affective ability linked to prosocial responding that emerges early in development^20^ and (b) stipulate the presence of a prosocial temperament/personality factor that is largely explained by genetics^23^. From a developmental perspective, sensitive responding to fear in others can be traced back into the first year of life. By around 7 months of age, infants pay increased attention to fearful facial expressions as shown in behavioral and event-related potential studies^24^. Critically, this biased attention to fear in others has been shown to vary as a function of infant temperament^25^ and genetic variation within neurotransmitter systems^26^. Together with the current findings, this raises the prospect that by measuring individual differences in responsiveness to fear in others during infancy, it might be possible to examine prosocial temperament/personality and predict altruistic behavior in children.

The current data also revealed fascinating culture-specific effects. As hypothesized on the basis of prior work^17^, Indian but not German children showed a greater sensitivity to context when processing emotional facial expressions and this context sensitivity was related to their altruistic behavior. Specifically, among Indian children, those children who were slower to orient to the fearful face presented in the context of eight neutral faces (and were thus more sensitive to context) showed increased altruistic behavior in the Dictator Game. This is a surprising effect given that prior work with adults in Western societies^19^ reported a general association between increased altruistic behavior and greater responsiveness (faster orienting) to fearful faces. However, this effect is in agreement with work showing that Asians typically engage in greater context-dependent perceptual processes by attending to the relation between the object and the context in which the object is placed and are slower to fixate on a focal object in a visual scene than Westerners^27^. Interestingly, our data do not show that Indian children are generally more sensitive to context but rather that this increased sensitivity to context in Indian children is specific to fearful (but not happy) faces presented in context. This selective effect may suggest that particularly when attending to distress cues in others, context plays a greater role in Indian culture. More specifically, those Indian children who are more strongly affected (slowed down) by context when detecting fearful faces also show increased altruistic behavior. One possibility is that Indian children who behave more altruistically are more prone to be affected by bystanders (context) in the case of seeing others in distress. In other words, being slowed down by the other faces surrounding the fearful (distress) face might represent sensitivity to other potential (adult) helpers being present. However, this is a speculative proposal and the exact reason why Indian children that behaved more altruistically were slowed down by context remains to be examined in future studies. Regardless of the direction of the context effect, the current finding points to a culture-specific association between context-sensitive fear processing and altruistic behavior that in Indian children operates in addition to the culture-independent association described above.

In summary, the current findings provide new insights into the developmental and cultural origins of altruism. In particular, our results demonstrate that across cultures, responsiveness to fear in others is linked to altruistic behavior in childhood. Moreover, the current data provide evidence for culture-specific patterns of context-sensitivity in these processes. These findings paint a rich picture emphasizing key affective, developmental, and cultural components that characterize the nature of altruism in humans.

## Methods

### Participants

The final sample consisted of 96 German and Indian children (mean age = 4.55 years; females = 43). An additional 10 preschool-aged children were also tested, but were excluded from the final sample due to insufficient eye-tracking and behavioral data. The German sample consisted of 43 children (mean age = 4.52 years; females = 20) recruited via a child research database based in Leipzig, which is a large city in Germany. The Indian sample consisted of 53 children (mean age = 4.51 years; females = 22) recruited from the junior kindergarten section of Shishuvan School, Matunga Central, which is a suburb of Mumbai, India. In both samples, children came from urban middle-class families with largely comparable educational backgrounds and access to health care. Note that while children in both countries came from middle class families, there are likely to be differences in terms of family income across countries. However, in this context it is important to emphasize that the resources used in the Dictator Game, namely stickers, are likely to be of similar accessibility and popularity across the two countries. All parents provided written informed consent prior to the study and the children were given a toy as a present after the session. The sample sizes in both countries were determined before testing and analysis to be larger than 40 (in each country), in order to get a representative distribution of altruistic behavior^28^. But the exact sample size in each country also depended on the availability of children within this particular and predefined age range for which we received informed consent from the parents. Note that the overall sample size and the sample size for each culture are considerably larger than in most experimental studies with children of that age.

### Stimuli

In Task 1, we used color photographs of happy, fearful, and neutral facial expressions taken from the previously validated FACES database (http://faces.mpib-berlin.mpg.de)^29^. We selected photographs from four actresses (age 19 to 30, ID-numbers 28, 48, 163, 182). These actresses were selected on the basis of high recognition rates shown by a group of adult raters^29^ and on the basis of their ethnicity. Two of the four actresses had a facial appearance, especially skin color, chosen to ethnically represent faces typically seen among South Asians. The remaining two actresses had a facial appearance, chosen to ethnically represent faces typically seen among Caucasians. The photographs were cropped such that only the face, but not the hair, and ears, was visible in order to focus children on the inner features (eyes, nose, and mouth) of the faces. The face stimuli were 14.5 cm (height) by 11.5 cm (width) in size. Regions of interest (ROIs) were created within Tobii Studio. ROIs comprised of the entire face region of the stimuli. Please note that, due to the privacy laws enforced by the FACES database, we are unable to show the stimuli (faces of actresses) presented in this study.

In Task 2, we used the same face stimuli as in Task 1. However, in contrast to Task 1, here emotional faces (happy or fearful) were presented in the context of eight neutral faces. The faces were presented in a 3 by 3 matrix, against a black background; with the emotional face (happy or fearful) appearing in any of eight locations in the 3 by 3 matrix expect the center location. The location at which the emotional face appeared in each trial was randomized and changed from one trial to the next. The matrix was 20 cm in height and 16 cm in width. Each individual facial stimulus in the matrix was 5 cm (height) by 3.5 cm (width) in size. Regions of interest (ROIs) were created within Tobii Studio. ROIs comprised of the entire matrix and every individual facial stimuli in the matrix.

### Eye-tracking procedure

The child sat on a chair approximately 60 cm away from a 17″ laptop screen. The region behind the computer monitor was a blank white washed wall to prevent any distractions for the child. A Tobii X2-60 compact eye tracker was set up at the bottom of the laptop screen in order to record the child’s looking behavior. Stimuli were presented through Tobii Studio (Version 3.2). Prior to stimulus presentation, a five-point calibration procedure was administered in order to ensure appropriate tracking of the children’s eyes. The German children were tested by a German experimenter in a testing room at a research institute, and the Indian children were tested by an Indian experimenter in a similar-sized and furnished testing room at Shishuvan School, Mumbai, India. Children were instructed to look at the laptop screen and pay attention to faces but were not asked to detect any particular emotion. The main aim of this instruction was to investigate the implicit capture of attention by emotional faces without giving the child explicit instructions to pay attention to emotional faces. Thus, we investigated natural scanning behavior rather than instructing the children to look for and detect specific emotions. In order to keep children attentive and motivated throughout, they were given a wireless mouse and asked to press the button whenever they saw an animated animal on the screen. Note that the button presses to the animations were not recorded, as they were not relevant for the actual experiment.

In Task 1, children viewed 12 trials, such that each actress presented each emotion once. The trial order was pseudo-randomized such that the same emotion and same actress did not appear twice in a row. The emotional faces (stimuli) appeared in the center of the screen and were presented for 2.5 s. Prior to the presentation of each face, a fixation item (asterisk) appeared at the center of the screen for 1.5 s in order to re-orient the child’s attention to the center of the screen. Four animations, showing an animated chicken, cat, dog, or lion, appeared once at random in between the trials. The duration of these animated clips varied from 1 to 3 s.

Task 2 was conducted after the completion of Task 1. Each child was given the same instructions prior to the start of Task 2 as in Task 1. In Task 2, children viewed 16 trials. In each trial, children saw an emotional face (either happy or fearful) in one of the eight positions in a 3 by 3 matrix. The emotional face never appeared in the center of the screen (matrix) because every trial was preceded by a fixation item (asterisk) shown in the center of the screen for 1.5 s. Each 3 by 3 matrix was presented for 6 s. As in Task 1, stimulus presentation was pseudo-randomized such that the same actress or emotion did not appear twice in a row. Six animations, showing a chicken, cat, dog, lion, lobster, or rattle appeared once in between trials. The duration of these animated clips varied from 1 to 3 s.

For data analysis, we extracted information regarding the latency to the first fixation and duration of looking at the emotional face. We focused our analysis on the latency for the first fixation and total fixation duration as dependent variables, as prior work suggests that these are sensitive measures for detecting individual variation in emotion processing and cultural variation in visual perception^18^,^30^. Because an initial analysis revealed no systematic effects for total fixation duration as a dependent variable, our analysis was focused on the latency for the first fixation measure. Critically, there were no differences in overall latency for the first fixation across cultures, indexing that there were no general differences in the way in which children in both cultures oriented towards the facial stimuli (see results section).

### Dictator Game

The Dictator Game followed the eye-tracking task. The experimenter placed five stickers in front of the child. The child was told that he or she could have all five stickers. Then the experimenter told the child about an unfamiliar peer in the adjoining room (gender and age of the other child were matched to the participant child) who had no stickers. The child was then asked whether he or she would be willing to share stickers with the peer. If the child agreed to share with the peer the experimenter asked how many of the stickers the child would like to share. The child was then asked to put the stickers he or she wanted to share in a box on the table in front of them. The instructions were given to the children by a native experimenter in their native language. Note that the Indian children grow up multi-lingually and the primary language of instruction spoken at the school where the experiments were conducted is English. The native Indian experimenter therefore instructed the children in English.

Please also note that the review board at the Max Planck Institute for Human Cognitive and Brain Sciences, Germany approved the protocol for the study and it was carried out in accordance with the provisions of the World Medical Association Declaration of Helsinki.

## Additional Information

**How to cite this article**: Rajhans, P. *et al*. Children’s altruistic behavior in context: The role of emotional responsiveness and culture. *Sci. Rep*. **6**, 24089; doi: 10.1038/srep24089 (2016).
